# The Therapeutic Effect of Botulinum Toxin Type A on Trigeminal Neuralgia: Are There Any Differences between Type 1 versus Type 2 Trigeminal Neuralgia?

**DOI:** 10.3390/toxins15110654

**Published:** 2023-11-14

**Authors:** Yan Tereshko, Mariarosaria Valente, Enrico Belgrado, Chiara Dalla Torre, Simone Dal Bello, Giovanni Merlino, Gian Luigi Gigli, Christian Lettieri

**Affiliations:** 1 Clinical Neurology Unit, Udine University Hospital, Piazzale Santa Maria della Misericordia 15, 33100 Udine, Italy; tereshko.yan@spes.uniud.it (Y.T.); giovanni.merlino@asufc.sanita.fvg.it (G.M.); christian.lettieri@asufc.sanita.fvg.it (C.L.); 2 Department of Medicine (DAME), University of Udine, Via Colugna 50, 33100 Udine, Italy; gigli@uniud.it; 3 Neurology Unit, Udine University Hospital, Piazzale Santa Maria della Misericordia 15, 33100 Udine, Italy; enrico.belgrado@asufc.sanita.fvg.it (E.B.); chiara.dallatorre@asufc.sanita.fvg.it (C.D.T.)

**Keywords:** trigeminal neuralgia, neuropathic pain, botulinum toxin type A, type 2 trigeminal neuralgia, type 1 trigeminal neuralgia, atypical trigeminal neuralgia

## Abstract

Background: Botulinum toxin type A is an effective treatment for trigeminal neuralgia. Moreover, its efficacy in type 2 trigeminal neuralgia and comparative studies between type 1 and type 2 trigeminal neuralgia (TN) still need to be improved. Methods: We treated 40 TN patients with onabotulinumtoxinA; 18 had type 1 TN, and 22 had type 2 TN. We compared the baseline pain score with the Visual Analogue Scale (VAS) and paroxysm frequency (number per week) at the baseline with those obtained at 1-month and 3-month follow-ups. Nonetheless, we compared the baseline Penn Facial Pain Scale with the scores obtained at the 1-month follow-up. Results: BoNT/A effectively reduced pain intensity and frequency at the 1-month and 3-month follow-ups. Moreover, the type 1 TN and type 2 TN groups had baseline pain scores of 7.8 ± 1.65 and 8.4 ± 1.1, respectively. Pain significantly improved (*p* < 0.001) in both groups to 3.1 ± 2.3 (type 1 TN) and 3.5 ± 2.3 (type 2 TN) at the 1-month follow-up and to 3.2 ± 2.5 (type 1 TN) and 3.6 ± 2.5 (type 2 TN) at the 3-month follow-up. There was no difference between the two groups (*p* 0.345). The baseline paroxysm frequencies (number per week) were 86.7 ± 69.3 and 88.9 ± 62.2 for the type 1 and type 2 TN groups, respectively; they were significantly reduced in both groups at the 1-month and 3-month follow-ups without significant differences between the two groups (*p* 0.902). The Pain Facial Pain Scale improved at the 1-month follow-up, and no significant differences were found between the two groups. There was a strong correlation between background pain and paroxysm pain intensity (r 0.8, *p* < 0.001). Conclusions: Botulinum toxin type A effectively reduced the pain, paroxysm frequency, and PFPS scores of type 1 and type 2 trigeminal neuralgia patients without statistically significant differences. Facial asymmetry was the only adverse event.

## 1. Introduction

Trigeminal neuralgia (TN), or *tic doloreux*, is a chronic pain syndrome typically characterized by recurrent paroxysmal episodes of brief severe unilateral pain in one or more branches of the trigeminal nerve [1,2,3]. The pain is mainly described as stabbing, shock-like, lancinating, or burning; innocuous stimuli can precipitate it, or it can be spontaneous. The estimated incidence of trigeminal neuralgia ranges from 4.3 to 26.8 cases per 100,000 person-year and increases with age [4,5,6,7]. The incidence is higher in women (2–3:1), and the mean age of onset is 53 years (24–93). Moreover, the ICHD-3 subclassified TN into three groups [3]. In classical TN, there is neurovascular compression of the trigeminal nerve root entry zone (REZ) associated with morphological changes in brain MRI; the proximal compression of the root entry zone by a blood vessel may start the process of focal demyelination and remyelination, with microvascular ischemic damage leading to axonal loss and demyelination [8,9]. Idiopathic TN requires normal brain MRI and neurophysiological studies. The secondary TN needs an underlying disease other than neurovascular compression to explain the symptoms; cerebellopontine angle tumors or Multiple Sclerosis frequently cause it. The tumors are often benign and compress the trigeminal root at its entry, causing demyelination similar to neurovascular compression [10,11]. In Multiple Sclerosis, there is significant inflammatory demyelination at the REZ [12,13]; however, central demyelinating lesions involving the intra-pontine trigeminal nucleus or the brainstem ipsilaterally to the affected side could also lead to TN [14,15]. Demyelination is a process that could lead to the genesis of neuropathic pain, lowering the excitability threshold and promoting ephaptic propagation towards nearby fibers since the myelin sheath is damaged [16,17,18,19]. The significant effect of sodium channel blockers suggests their involvement in the pathogenesis of TN; there is also evidence of abnormal expression of Nav1.7, Nav1.3, and Nav1.8 [20]. Clinically, TN can be characterized by the sole presence of paroxysmal pain in the territory of the branch or branches involved, defined as typical or type 1 TN, or by continuous pain superimposed upon the painful paroxysmal attacks, described as atypical or type 2 TN [21,22,23,24]. Quality of life is frequently impaired on a multidimensional level (socioeconomic, physical, mental) due to the intense pain and the frequent paroxysms, which are either spontaneous or triggered [25,26,27]. Carbamazepine and oxcarbazepine are the most used and effective treatment options for TN; almost 90% of patients have initial pain relief, but 40% have significant adverse events, determining withdrawal of the drug [28]. Other medical treatment options include baclofen, lamotrigine, gabapentin, pregabalin, levetiracetam, pimozide, clonazepam, phenytoin, eslicarbazepine, and vixotrigine, but the literature regarding their efficacy has less evidence [29]. Refractory TN patients may benefit from more invasive approaches, such as radiofrequency ablation, chemodenervation, balloon compression, cryotherapy, radiosurgery, peripheral neurectomy, or microvascular decompression. Type 2 TN patients tend to have less favorable outcomes and more adverse events with radiofrequency ablation, microvascular decompression, and radiosurgery when compared to type 1 TN [30,31,32,33,34,35]. Botulinum toxin type A (BoNT/A) is an effective and safe treatment for TN, with transitory facial asymmetry as the primary adverse event [36]. The role of BoNT/A in TN is in the setting of TN that is refractory to conventional oral therapies; however, 10–43% of the patients treated with this drug still do not respond [37]. Although the exact mechanisms of action of BoNT/A on neuropathic pain are still unclear, it has been demonstrated that this drug has selective entrance into capsaicin-sensitive sensory neurons, and the TRPV1-expressing neurons in both the peripheral and central nervous systems have a high affinity to this drug [38]. Interestingly, BoNT/A reduces the expression of TRPV1 in the sensory ganglia of damaged nerves [39,40,41] and reduces the release of CGRP of cultured trigeminal cells [42,43]. Nonetheless, the effects of Botulinum Toxin Type A effect on pain tend to ameliorate depression, anxiety, and the sleep of TN patients [44]. The literature is scarce regarding the use of BoNT/A for type 2 TN, and its efficacy is not well established when compared to type 1 TN. Our study aims to evaluate the response of TN patients to BoNT/A and to compare the therapeutical response of type 1 and type 2 TN patients to BoNT/A. 

## 2. Results

Forty TN patients were treated with BoNT/A; the mean age was 63.4 ± 18.4 years old, and 67.5% were female. Eighteen had type 1 TN, whereas twenty-two had type 2 TN. The mean disease duration was 5.8 ± 4.6 years; the type 2 TN group had a longer duration of the disease and a greater mean age than the type 1 TN group. Seventeen out of forty patients (42.5%) were on CBZ or OXC, and 42.5% were on gabapentinoids. The left side was involved in 47.5% of cases. Twenty-two patients had idiopathic etiologies, and the remaining eighteen patients had a classical form of TN; no cases of secondary TN were present in our sample. Twenty-two and eighteen patients had only one or two branches involved, respectively. The mean BoNT/A doses were 29.7 ± 11.4 U and 29.3 ± 11.1 U for the type 1 and type 2 TN groups, respectively. See Table 1 for detailed demographics and clinical features of all the TN patients.

**Figure 1:**
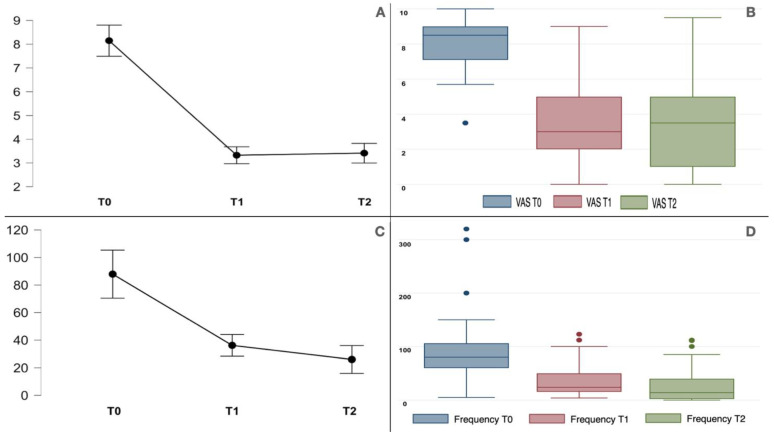
(**A**,**B**) The plot line (with standard error bars) and the boxplot for VAS of all the TN patients studied at the baseline (T0) and at 1-month (T1) and 3-month (T2) evaluations. There was a significant improvement in pain intensity throughout the follow-ups (F_1.2,48.8_ = 128.2, *p* < 0.001). (**C**,**D**) The plot line (with standard error bars) and the boxplot for paroxysm frequency (number per week) of all the TN patients studied at the baseline (T0) and at 1-month (T1) and 3-month (T2) evaluations; this variable improved significantly (F_1.0,40.4_ = 28.9, *p* < 0.001). The boxplots are represented by the median, the interquartile range, and the minimum and maximum scores (whiskers); the dots represent the outliers.

**Figure 2:**
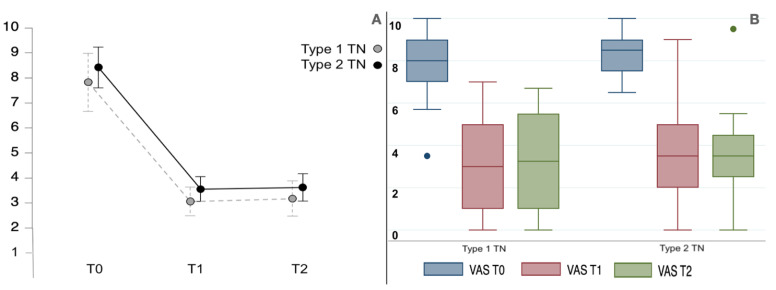
(**A**,**B**) The plot line (with standard error bars) and the boxplot for VAS of type 1 and type 2 TN patients studied at the baseline (T0) and at 1-month (T1) and 3-month (T2) evaluations. Repeated measures ANOVA was statistically significant (F_1.2,47.5_ = 123.4, *p* < 0.001). The boxplots are represented by the median, the interquartile range, and the minimum and maximum scores (whiskers); the dots represent the outliers.

**Figure 3:**
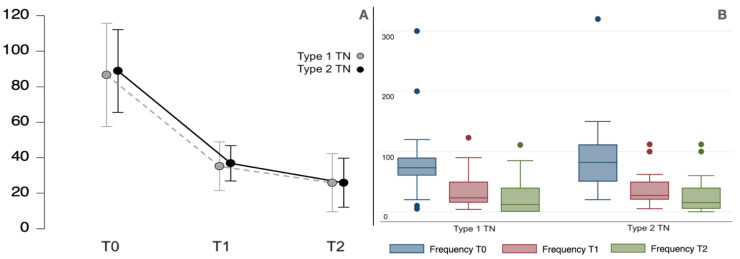
(**A**,**B**) The plot line (with standard error bars) and the boxplot for paroxysm frequency (number per week) of type 1 and type 2 TN patients studied at the baseline (T0) and at 1-month (T1) and 3-month (T2) evaluations. There was a significant reduction in frequency in both groups (F_1.0,39.4_ = 27.8, *p* < 0.001). The boxplots are represented by the median, the interquartile range, and the minimum and maximum scores (whiskers); the dots represent the outliers.

All the TN patients improved in their VAS (F_1.3,48.8_ = 128.3, *p* < 0.001) and paroxysm frequency (F_1.0,40.4_ = 28.9, *p* < 0.001), as shown in Figure 1. The baseline VAS score for the type 1 TN group was 7.8 ± 1.6; it improved at the 1-month (3.1 ± 2.2) and 3-month (3.2 ± 2.5) follow-ups (see Figure 1). The type 2 TN group had a baseline VAS score of 8.4 ± 1.1 that reduced to 3.5 ± 2.3 and 3.6 ± 2.5 at the 1-month and 3-month follow-ups, respectively. The repeated measures ANOVA was statistically significant for VAS score reduction in both groups (F_1.3,47.5_ = 123.5, *p* < 0.001). Both groups had their paroxysm frequencies (number of episodes per week) significantly reduced (F_1.0,39.4_ = 27.8, *p* < 0.001) at the 1-month and 3-month follow-ups (see Table 2 for detailed post hoc comparison statistics within the groups performed with Bonferroni correction). The two groups had no difference in VAS (F_1,38_ = 0.9, *p* 0.345) and paroxysm frequency (F_1,38_ = 0.01, *p* 0.902). The graphical representations of the data are shown in Figure 2 and Figure 3. Moreover, the background pain of the type 2 TN group improved from 5.9 ± 1.9 to 2.1 ± 1.9 at the 3-month follow-up (*p* < 0.001).

**Figure 4:**
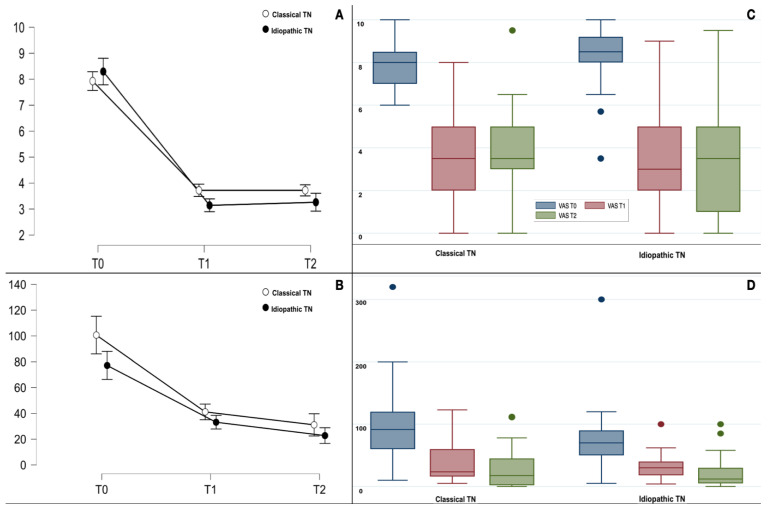
(**A**,**C**) The plot line (with standard error bars) and the boxplot for paroxysm pain (VAS) in classical and idiopathic TN at the baseline (T0) and at 1-month (T1) and 3-month (T2) evaluations. (**B**,**D**) The plot line (with standard error bars) and the boxplot for paroxysm frequency (number per week) at the baseline (T0) and at 1-month (T1) and 3-month (T2) evaluations. The boxplots are represented by the median, the interquartile range, and the minimum and maximum scores (whiskers); the dots represent the outliers.

Seven patients out of forty (17.5%) were pain-free at the 1-month follow-up; four had type 1 TN, and three had type 2 TN. Six patients were pain-free at the 3-month follow-up (four had type 1 TN, and the remaining two had type 2 TN). The treatment was effective (>50% pain reduction) for 25 patients (62.5%) at the 1-month follow-up; among them, 11 patients had type 1 TN (27.5%) and 14 had type 2 TN (35%). At the 3-month follow-up, the treatment was considered effective for 24 patients (60%); 13 had type 2 TN (32.5%), and 11 had type 1 TN (27.5%). The mean durations of BoNT/A were 3.4 ± 1.4 and 3.5 ± 1.5 months in the type 1 and type 2 groups, respectively; there was no statistical difference between the two groups (*p* 0.944). The overall mean duration was 3.5 ± 1.5 months; the maximum duration of BoNT/A was up to 6 months. The PFPS total scores and every item of this scale improved significantly in both groups (see Table 3); there was no significant difference between the two groups (see Table 4). A graphical representation of the mean scores of all the PFPS items at the baseline and the 1-month follow-up is shown in Figure 4.

PGIC 3-month reports were as follows (see Table 5): four patients reported that the treatment did not improve their pain (PGIC 3/7; “A little better, but no noticeable change”), and one patient reported that the improvement was not satisfying (PGIC 4/7; “Somewhat better, but the change has not made any real difference”); three patients reported a mild improvement (PGIC 5/7; “Moderately better, and a slight but noticeable change”), while twenty-six patients signaled a significant improvement (PGIC 6/7; “Better, and a definite improvement that has made a real and worthwhile difference”). Six patients improved more (7/7; “A great deal better, and a considerable improvement that has made the difference”). 

The presence of type 2 TN did not predict a more favorable or less favorable response (<50% VAS reduction) to the treatment with BoNT/A (OD 1.4; C.I: 95% 0.3–3.3, *p* 0.908). In addition, idiopathic vs. classical etiology (OD 1.1; C.I: 95% 0.4–5.1, *p* 0.591) did not predict the outcome. In fact, as shown in Figure 4, the classical and idiopathic TN groups improved significantly in paroxysm intensity (F_1.3,46.6_ = 121.5, *p* < 0.001) and frequency (F_1.0,38.4_ = 27.1, *p* < 0.001), without significant differences (F_1.0,37.0_ = 0.2, *p* 0.683 for paroxysm intensity and F_1.0,37.0_ = 1.7, *p* 0.200 for paroxysm frequency).

At the 1-month follow-up, the responder group (>50% VAS reduction) improved in total PFPS score from 116.0 ± 18.7 to 37.1 ± 29.6 (*p* < 0.001); the PFPS score (only considering the interference with daily facial activities score) improved from 88.3 ± 18.8 to 29.3 ± 23.6 (*p* < 0.001). The total PFPS score of the non-responder group (<50% VAS reduction) also improved from 115.6 ± 41.0 to 67.6 ± 42.6 (*p* <0.001); the PFPS score, only considering the interference with daily facial activities score, improved from 87.6 ± 37.0 to 50.3 ± 36.5 (*p* < 0.001). We compared the mean PFPS score reductions (interference with daily facial activities) of the responder and non-responder groups, and the difference was not significant (59.0 ± 23.9 vs. 37.4 ± 30.4; *p* 0.065).

**Figure 5:**
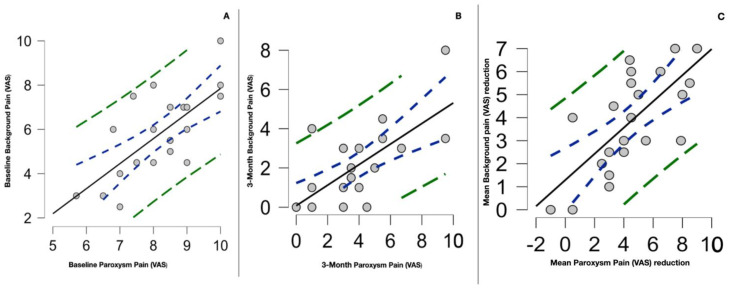
(**A**) Scatter plot of the significant correlation between baseline paroxysm pain intensity and baseline background pain intensity of type 2 TN patients. Analysis was performed with Spearman’s correlation test (r 0.8, *p* < 0.001). (**B**) The same correlation remained statistically significant at 3-month evaluation (r 0.7, 0.4–0.9, *p* < 0.001). (**C**) Scatter plot significantly correlating the mean reductions in paroxysm pain and background pain (r 0.7, 0.4–0.9; *p* < 0.001). The dotted blue curve represents the 95% confidence interval, while the green dotted curve represents the 95% prediction interval.

**Figure 6:**
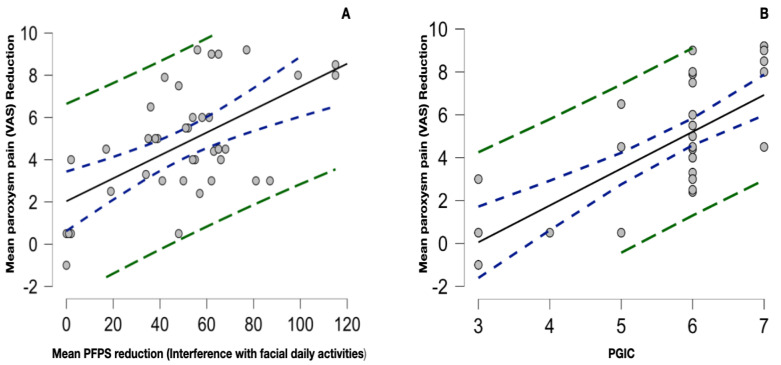
(**A**) Scatter plot of the significant correlation between the mean paroxysm pain reduction and the mean PFPS score reduction (interference with daily facial activities only); r 0.6, 0.3–0.8, *p* < 0.001. (**B**) Significant correlation between the PGIC and the mean paroxysm pain reduction; r 0.7, 0.5–0.8, *p* < 0.001. The dotted blue curve represents the 95% confidence interval, while the green dotted curve represents the 95% prediction interval.

Interestingly, there was a strong correlation between the baseline paroxysm pain intensity and the baseline background pain intensity of type 2 TN patients (r 0.8, 0.4–0.9 95% CI; *p* < 0.001), as shown in Figure 5. There was no correlation between baseline background pain intensity and baseline paroxysm frequency (r −0.1, −525–0.3 95% CI; *p* 0.555), disease duration (r −0.2, −0.6–0.2 95% CI; *p* 0.293), or age (r −0.01, −0.4–0.4 95% CI; *p* 0.961). The correlation between background and paroxysm pain at the 3-month evaluation remained statistically significant (r 0.7, 0.4–0.9, *p* < 0.001). There was also a significant correlation between the mean paroxysm pain and background pain reduction (r 0.7, 0.4–0.9; *p* < 0.001). The mean paroxysm pain reduction (VAS) positively correlated with the mean PFPS score (interference with daily facial activities) reduction (r 0.6, 0.3–0.8, *p* < 0.001); the mean paroxysm pain reduction (VAS) also positively correlated with the PGIC (r 0.7, 0.5–0.8, *p* < 0.001), as shown in Figure 6. 

Facial asymmetry was reported by fourteen patients (six patients in the type 2 TN group and eight patients in the type 1 TN group). This adverse event was reported to be mild to moderate, lasting 2–3 weeks to 1–2 months. There were no other adverse events reported. 

## 3. Discussion

Type 2 TN is more frequent in women and is more frequently associated with sensory abnormalities than type 1 TN [45,46]. Continuous or near-continuous background pain between paroxysmal attacks, which is present in type 2 TN, is unrelated to the etiology and occurs in idiopathic, classical, or secondary forms [46]. The quality of pain is frequently dull, burning, or tingling, and the distribution coincides with that of the paroxysmal pain [47,48,49]. There are fluctuations in intensity, remission periods, and recurrences that follow paroxysmal pain. However, paroxysmal and continuous pain may improve independently after surgical treatment [50,51,52]. The pathophysiology underlying the background pain in type 2 TN is unclear; a recent study where type 1 and type 2 TN patients were investigated via pain-related evoked potential (PREP) and nociceptive blink reflex (NBR) showed that type 2 TN patients had shorter latencies and higher amplitudes of PREP when compared to type 1 TN patients [53]. This implies the facilitation of central pain processing, which is determined by reorganizing and overactivating the supraspinal trigeminal nociceptive system, which probably involves the thalamus or the cingulate cortex. It has been implied that background pain could derive from a pronounced involvement of unmyelinated fibers (C fibers) at the trigeminal sensory root; these fibers convey slow pain with a burning or dull quality [54]. Moreover, in a cohort of 158 TN patients, among 79 with type 2 TN, 18% of them had background pain from the onset of TN, preceding the presence of paroxysmal pain, while 72% developed it after a mean time of 1.5 years [55]. The type 2 TN group had lower responses to sodium channel blockers when compared to type 1 TN (81% vs. 98%), probably due to their mechanism of blocking the high-frequency discharges that underlie the typical paroxysmal pain [55]. A recent study performed with a 3T brain MRI showed that the trigeminal root was more severely atrophic in patients with type 2 TN [56]. Since the trigeminal sensory root is more severely atrophic in type 2 TN patients, some authors have suggested that this phenomenon is related to axonal loss and abnormal activity in denervated trigeminal neurons [56]. Type 2 TN patients have lower pain freedom and duration success rates with stereotactic radiosurgery [33,34]. Microvascular decompression has a higher rate of success for type 1 TN when compared to type 2 TN; the long-term pain-free rates are 74% and 60%, respectively [35]. BoNT/A has been demonstrated to be an effective and safe therapy for TN; however, the literature on its efficacy for type 2 TN is still scarce. Type 2 TN was treated with BoNT/A with significant efficacy in two case reports [57,58], in one open-label study with both type 1 and type 2 patients [59], and in a study with ten type 2 TN patients [60]. Only one retrospective study [61] performed a subanalysis and compared type 1 and type 2 in a cohort of patients with idiopathic and secondary TN due to multiple sclerosis; they reported higher efficacy among the type 2 TN patients when compared to type 1 TN patients (OD 0.211, 0.046–0.98; *p* 0.047) on the univariate logistic regression; however, multivariate logistic regression was not performed.

In our study, BoNT/A treatment was effective overall (>50% pain reduction) for 62.5% and 60% of patients at the 1-month and 3-month follow-ups, respectively; these data are in line with the current literature [37]. Moreover, seven and six patients were pain-free at the 1-month and 3-month follow-ups. Specifically, the treatment was effective for 61.1% of type 1 TN and 63.6% of type 2 patients at the 1-month follow-up. The data were similar at the 3-month follow-up since 61.11% of type 1 and 59.1% of type 2 TN patients had a pain reduction of >50% at the 3-month follow-up. The only adverse event was facial asymmetry, which was mild and transient in all the affected patients. The type 1 and type 2 TN groups improved in pain intensity, paroxysm frequency, and PFPS similarly without statistically significant differences. Moreover, in both types of TN, the classical and idiopathic forms had similar results in terms of efficacy. The duration of the effect of BoNT/A was also similar in the two groups. There was a significant positive correlation between the mean paroxysm pain reduction (VAS) and the mean PFPS score reduction (interference with daily facial activities); the same significant correlation was found with the PGIC. These data suggest that the improvement in pain intensity also leads to improvement in quality of life. The mean PFPS reduction (interference with daily facial activities) was similar among responders and non-responders; this suggests that despite the failure in reducing the paroxysmal pain intensity by at least 50% at the 1-month follow-up, the quality of life improved significantly, and this must be taken into consideration in the decisional process. Interestingly, there was a strong correlation between background and paroxysm pain intensity in type 2 patients, both at the baseline and at the 3-month evaluation; this could suggest a correlation between the entity of the dysfunction of the fibers that convey paroxysmal pain (A-delta and A-beta fibers) and continuous pain (C fibers). The correlation between the mean reductions in paroxysm pain and background pain suggests an effect of BoNT/A on both pain phenotypes (paroxysmal and continuous pain), thus suggesting antinociceptive mechanisms that act on the three different fibers involved (A-beta, A-delta, and C fibers). Although the exact mechanisms of action of BoNT/A on neuropathic pain are still unclear, researchers have demonstrated selectivity for the afferents expressing the TRPV1 (C and A-delta fibers) of capsaicin-sensitive neurons, interfering with its expression on the surface of the plasma membrane of the neuronal ganglia and neurons of the central nervous system [38,62]. BoNT/A has a central antinociceptive effect, probably due to the retrograde axonal and trans-synaptic transport; the injection of BoNT/A into a rat whisker pad showed the presence of cleaved SNAP25 in the trigeminal nucleus pars oralis and caudalis [63,64,65]. An indirect effect of BoNT/A in distant sensory regions of orofacial pain induced by formalin has also been described; c-Fos expression in the PAG and locus coeruleus was diminished, suggesting a pain modulatory effect on the ascending sensory areas [39,40,41]. The reduced expression of TRPV1 following BoNT/A injections was demonstrated in sensory neurons projecting from the dura mater, corroborating the theory of axonal retrograde transport and trans-synaptic translocation between different sensory neurons [42,63,65,66]. In addition, BoNT/A inhibits the release of CGRP and substance P, which are involved in peripheric and central sensitization [67,68,69,70]. Since type 1 and type 2 TN have both peripheral (A-delta, A-beta, and C fibers) and central involvement, BoNT/A could act in both systems, modulating the pain transmission of peripheral fibers and regulating the central nociceptive system; both types of TN can benefit from BoNT/A injections.

### Limitations of the Study

Our study has some limitations. The first one is the relatively small sample and the short follow-up. Since this was a single-center study, there could be a bias in patient selection, implying that the sample may not represent the entire TN population. 

## 4. Conclusions

Botulinum toxin type A is an effective and safe treatment for TN patients; the only adverse event was facial asymmetry. Type 1 and type 2 TN had similar responses to the treatment with BoNT/A in pain intensity, paroxysm frequency, and PFPS, without statistically significant differences. Larger studies are needed to confirm our results.

## 5. Methods

### 5.1. Study Design and Participants

This is an open-label, single-center, longitudinal study. Forty patients >18 years old were recruited from our tertiary headache outpatient clinic from January 2018 to August 2022 (Clinical Neurology Unit; Ospedale S. Maria della Misericordia, Udine, Italy). The patients fulfilled the criteria for trigeminal neuralgia, and the presence of type 1 (ICHD-3 13.1.1.1.1 or 13.1.1.3.1) and type 2 (ICHD-3 13.1.1.1.2 or 13.1.1.3.2) TN was assessed according to ICHD-3 [3]. BoNT/A treatment was proposed to all consecutive TN patients who were refractory to oral medical therapy, for whom the oral medications were contraindicated, or to patients reluctant to perform surgery or try other conventional oral medications. None of the patients who satisfied the inclusion criteria refused to consent to treatment, and none of them had clinical conditions that contraindicated the treatment with BoNT/A (neuromuscular junction diseases, motor neuron diseases, or infection and dermatitis in the affected region). Other concomitant preventive oral medications (carbamazepine, oxcarbazepine, pregabalin, or gabapentin) for TN were permitted if the therapy was present for at least three months before the BoNT/A treatment and remained unchanged during the follow-up. Twenty-three patients were on CBZ or OXC (eleven type 1 TN and twelve type 2 TN), and seventeen were on pregabalin or gabapentin (seven type 1 TN and ten type 2 TN). Patients’ Global Impression of Change (PGIC) was assessed at a 3-month follow-up. Visual Analogue Scale (VAS), weekly paroxysm frequency, and Penn Facial Pain Scale (PFPS) were evaluated at the baseline and the 1-month and 3-month follow-ups. For type 2 TN, we assessed the background pain at the baseline and the 3-month follow-up. The patients were instructed to fill out a diary in which they reported the paroxysm frequency, pain intensity (paroxysm and/or background pain), and adverse events. The patients reported the duration of the effects of BoNT/A during the following months after the injections by telephone or during the outpatient visit.

### 5.2. Ethics

This study was conducted following the Declaration of Helsinki and approved by the Institutional Review Board of the University of Udine (IRB-DAME; Prot IRB: 150/2022). All patients gave written consent for the treatment with BoNT/A and for their clinical data to be used for research purposes.

### 5.3. Treatment with BoNT/A

A 100 U vial of onabotulinumtoxinA (BOTOX^®^) was reconstituted with 1 mL of sterile sodium chloride at 0.9% to achieve a dilution of 100 U/mL. The injections were performed with a 30-gauge 0.3 × 8 mm needle. A total of 5–2.5 U was injected intradermally or subcutaneously in the region of pain (“follow the pain” approach) and along the course of the trigeminal branches involved. For the treatment of the V1 branch, we injected 2.5–5 U above the eyebrow, in the mid-frontal and upper-frontal regions of the affected side (separated 2 cm horizontally and 1 cm vertically from each other) to form a rectangle; the supraorbital or supratrochlear exit zones were treated with 5 U if there was a trigger point. For the V2 and the V3 branches, the treatment was performed following the course of the nerves; we injected 5–2.5 U per site with a total of 5–6 points per side, separated by 1.5–2.5 cm. We applied a higher dose (5 U) in the terminal branches’ trigger point regions and exit sites.

### 5.4. PFPS, PGIC, and VAS

The Penn Facial Pain Scale (PFPS) is a multidimensional scale used to evaluate facial pain severity (4 items: least, worst, average, and pain right now) and pain interference with daily facial activities (14 items). The patient gives a score from 0 to 10 for every item, and the total score is calculated from the sum of each item [71]; obtaining a separate score for the pain and interference with daily facial activities domains is also possible. The Patients’ Global Impression of Change (PGIC) is a validated questionnaire for chronic pain in which patients rate the level of change from their baseline condition. It reflects the patients’ impression of the effect of a therapy [72]. The 10 cm Visual Analogue Scale is used to assess pain severity [73].

### 5.5. Endpoints

The study’s primary endpoint was to assess the efficacy of BoNT/A based on the pain intensity and paroxysm frequency during the follow-up. The treatment was effective when pain intensity (VAS) improved >50% at 1-month and 3-month follow-ups. 

The secondary endpoint compared the responses in type 1 and type 2 TN based on the frequency of paroxysms (number per week), pain intensity (VAS), and PFPS. 

The tertiary endpoint compared the responses in classical and idiopathic TN based on pain intensity (VAS) and frequency of paroxysms (number per week).

### 5.6. Statistical Analysis

The descriptive analysis of the sample was performed using mean ± SD for continuous variables and percentages for categorical variables. A *t*-test, when the data distribution was normal, or a Mann–Whitney test, when the data distribution was not normal, was used to compare continuous variables. A chi-square test was used to compare categorical variables. A Shapiro–Wilk test was used to assess the normal distribution of data. We calculated the power of the study with G*power 3.1 (for ANOVA repeated measures, within and between interactions); we set an effect size of 0.25, an *α*-error of 0.05, two groups, three measurements, forty subjects, and a correlation among repeated measures of 0.5. The preplanned power of the study was 0.9. Comparison between groups was performed with a *t*-test or Mann–Whitney test as appropriate for the PFPS. A paired *t*-test, when the data had a normal distribution, or a Wilcoxon test, when the distribution was not normal, was used to compare the PFPS and background pain data at the baseline and 1-month and 3-month follow-ups, respectively. Repeated measures ANOVA was used to investigate the changes in pain intensity (VAS) and paroxysm frequency (number per week) at the baseline and 1-month and 3-month follow-ups. Because Mauchly’s test of sphericity was significant, we used the Greenhouse–Geisser correction. Repeated measures ANOVA was also used to compare the differences in mean scores for the type 1 and type 2 TN groups. A Bonferroni post hoc test was used to compare the means at different follow-up times. For the analysis of the predictive factors for response (>50% pain reduction at three months), we conducted a univariate logistic regression analysis considering the type of TN (type 1 and type 2) and the etiology (idiopathic and classical); the odds ratio (OR), the 95% confidence interval (CI), and the level of significance (*p*) are presented. Repeated measures ANOVA was used to investigate the changes in pain intensity and paroxysm frequency in idiopathic and classical TN at the baseline and at 1-month and 3-month follow-ups. Correlation analysis was performed with Spearman’s test. All analyses used Stata/SE (version 15.1, StataCorp, College Station, TX, USA) for Mac OS. All 2-tailed statistical significance levels were set at *p* < 0.05. 

## Figures and Tables

**Table 1:** The demographics and clinical characteristics of all the TN patients and the subdivision of type 1 and type 2 TN patients. Legend: TN, trigeminal neuralgia; CBZ, carbamazepine; OXC, oxcarbazepine; V1, ophthalmic branch; V2, maxillary branch; V3, mandibular branch; BoNT/A, botulinum toxin type A. A *t*-test or a Mann–Whitney test was used, as appropriate, to compare continuous variables between type 1 and type 2 TN; a chi-square test was used for qualitative variables.

Variable	TN	Type 1 TN (Mean ± SD or N°, /%)	Type 2 TN (Mean ± SD or N°, /%)	*p*-Value
Number of patients	40	18	22	
Age	63.4 ± 18.4	59.9 ± 20.1	66.2 ± 16.9	0.282
Female sex	27/40 (67.5%)	13/18 (72.2%)	14/22 (63.6%)	0.564
TN duration (years)	5.8 ± 4.6	4.2 ± 3.9	7.0 ± 4.8	0.027
Previous oral prophylaxis	1.3 ± 1.2	0.9 ± 1.0	1.6 ± 1.1	0.051
**TN characteristics**				
Left side involved	19/40 (47.5%)	9/18 (50.0%)	10/22 (45.5%)	0.775
Idiopathic TN	22/40 (55.0%)	11/18 (61.1%)	11/22 (50.0%)	0.482
Classical TN	18/40 (45.0%)	7/18 (38.9%)	11/22 (50.0%)	0.537
Only V1 involved	4/40 (10.0%)	0/18 (0%)	4/22 (18.2%)	0.114
Only V2 involved	10/40 (25.0%)	6/18 (33.3%)	4/22 (18.2%)	0.300
Only V3 involved	8/40 (20.0%)	3/18 (16.7%)	5/22 (22.7%)	0.709
V1 + V2 involved	6/40 (15.0%)	4/18 (22.2%)	2/22 (9.1%)	0.381
V2 + V3 involved	12/40 (30.0%)	5/18 (27.8%)	7/22 (31.8%)	0.781
BoNT/A dose	29.5 ± 11.1	29.7 ± 11.4	29.3 ± 11.0	0.967

**Table 2:** Comparison of VAS and frequency values at T0, T1, and T2. The statistics were performed with repeated measures ANOVA and Bonferroni post hoc tests. A paired *t*-test (normal data distribution) was performed to compare background pain at baseline with the score obtained at the 3-month follow-up; the *p*-value was set at 0.05 for both tests. Legend: TN, trigeminal neuralgia; T =, baseline assessment; T1, 1-month follow-up; T2, 3-month follow-up; *, T0 vs. T1; ** T0 vs. T2; ***, T1 vs. T2; NS, not significant.

	T0	T1	T2	*p (Post Hoc Comparison)*
**All TN Patients**				
Pain (VAS)	8.1 ± 1.4	3.3 ± 2.3	3.4 ± 2.4	*^,^ ** < 0.001 *** NS
Frequency	87.9 ± 64.6	36.2 ± 29.5	26.0 ± 31.9	*^,^ ** < 0.001 *** NS
**Type 1 TN group**				
Pain (VAS)	7.8 ± 1.6	3.1 ± 2.3	3.2 ± 2.5	*^,^ ** < 0.001 *** NS
Frequency	86.7 ± 69.3	35.2 ± 32.3	26.0 ± 34.4	* 0.002 ** < 0.001 *** NS
**Type 2 TN group**				
Pain (VAS)	8.4 ± 1.1	3.5 ± 2.3	3.6 ± 2.5	*^,^ ** < 0.001 *** NS
Frequency	88.9 ± 62.2	36.9 ± 27.8	26.0 ± 30.7	*^,^ ** < 0.001 *** NS
Background pain (VAS)	5.9 ± 1.9		2.1 ± 1.9	<0.001

**Table 3:** The means and SDs of all the items of the PFPS and the total scores at T0 and T1 for type 1 TN patients, type 2 TN patients, and all the TN patients of the study. A *t*-test or a Mann–Whitney test (when the data distribution was not normal) was used to compare; significance was determined with a *p*-value of 0.05.

	T0 Type 1 TN	T1 Type 1 TN	*p*	T0 Type 2 TN	T1 Type 2 TN	*p*	T0 TN	T1 TN	*p*
General activity	7.5 ± 2.3	2.7 ± 3.0	**<0.001**	7.8 ± 2.0	3.0 ± 2.7	**<0.001**	7.7 ± 2.1	2.9 ± 2.8	**<0.001**
Mood	7.6 ± 2.6	2.7 ± 2.9	**<0.001**	8.4 ± 2.3	3.0 ± 3.0	**<0.001**	8.0 ± 2.5	2.9 ± 2.9	**<0.001**
Walking	2.9 ± 3.9	0.6 ± 1.9	**0.033**	2.5 ± 3.8	0.8 ± 2.2	**0.022**	2.7 ± 3.8	0.7 ± 2.1	**0.002**
Work	5.6 ± 3.1	2.3 ± 2.7	**0.002**	6.2 ± 3.3	2.3 ± 2.7	**<0.001**	5.9 ± 3.2	2.3 ± 2.7	**<0.001**
Relationship	6.6 ± 2.6	2.1 ± 2.6	**<0.001**	7.0 ± 2.8	3.1 ± 2.6	**<0.001**	6.8 ± 2.7	2.7 ± 2.6	**<0.001**
Sleep	4.8 ± 3.3	2.1 ± 2.8	**0.004**	5.5 ± 3.9	1.5 ± 2.4	**<0.001**	5.2 ± 3.6	1.8 ± 2.5	**<0.001**
Enjoyment of life	7.4 ± 2.3	2.9 ± 3.1	**<0.001**	8.2 ± 1.4	3.3 ± 2.3	**<0.001**	7.9 ± 1.8	3.1 ± 2.7	**<0.001**
Eat	6.0 ± 3.0	2.4 ± 2.9	**0.002**	6.9 ± 3.7	3.0 ± 3.0	**<0.001**	6.5 ± 3.4	2.8 ± 3.0	**<0.001**
Touch your face	5.9 ± 3.4	3.7 ± 3.0	**<0.001**	7.6 ± 3.1	3.1 ± 3.2	**<0.001**	6.9 ± 3.3	3.4 ± 3.1	**<0.001**
Brush your teeth	5.6 ± 3.5	3.1 ± 3.1	**0.002**	6.5 ± 4.0	3.5 ± 3.2	**0.001**	6.1 ± 3.7	3.3 ± 3.1	**<0.001**
Smile or laugh	5.2 ± 3.6	2.7 ± 2.9	**0.003**	5.1 ± 4.0	2.6 ± 3.1	**0.002**	5.1 ± 3.8	2.7 ± 3.0	**<0.001**
Talk	5.6 ± 3.8	1.8 ± 2.3	**<0.001**	6.1 ± 3.8	3.5 ± 2.9	**<0.001**	5.9 ± 3.8	2.8 ± 2.7	**<0.001**
Open your mouth wide	5.7 ± 3.5	2.6 ± 2.4	**<0.001**	5.3 ± 4.0	2.6 ± 3.2	**0.002**	5.5 ± 3.8	2.6 ± 2.8	**<0.001**
Eat hard food	7.5 ± 2.6	4.2 ± 3.8	**0.004**	8.4 ± 2.4	5.0 ± 3.1	**<0.001**	8.0 ± 2.5	4.6 ± 3.4	**<0.001**
Worst NRS	9.3 ± 1.7	4.7 ± 3.4	**0.001**	9.6 ± 1.0	5.2 ± 2.7	**<0.001**	9.5 ± 1.4	5.0 ± 3.0	**<0.001**
Least NRS	5.3 ± 2.2	2.1 ± 1.9	**<0.001**	6.0 ± 2.1	2.3 ± 2.1	**<0.001**	5.7 ± 2.2	2.2 ± 2.0	**<0.001**
Mean NRS	7.4 ± 1.5	3.1 ± 2.3	**<0.001**	7.9 ± 1.4	3.5 ± 2.3	**<0.001**	7.7 ± 15	3.3 ± 2.3	**<0.001**
Pain now	4.4 ± 3.6	1.0 ± 2.2	**0.006**	5.5 ± 2.7	1.7 ± 2.5	**<0.001**	5.0 ± 3.1	1.4 ± 2.4	**<0.001**
**PFPS Total score**	110.3 ± 30.0	46.4 ± 37.5	**<0.001**	120.3 ± 29.5	53.1 ± 39.6	**<0.001**	115.8 ± 29.8	50.1 ± 38.3	**<0.001**

**Table 4:** Comparison of the means of items of the PFPS and the total scores at the baseline and at 1-month follow-up. Moreover, the means of reduction was also compared between the two groups. There was no difference between the two groups. A *t*-test or a Wilcoxon test (when the data distribution was not normal) was used to compare; significance was determined with a *p*-value of 0.05.

	T0 Type 1 TN	T0 Type 2 TN	*p*	T1 Type 1 TN	T1 Type 2 TN	*p*	Mean Reduction Type 1 TN	Mean Reduction Type 2 TN	*p*
**PFPS total**	110.3 ± 30.0	120.3 ± 29.5	0.293	46.4 ± 37.5	53.1 ± 39.6	0.549	63.3 ± 38.9	67.3 ± 32.1	0.764
General activity	7.5 ± 2.3	7.8 ± 2.0	0.646	2.7 ± 3.0	3.0 ± 2.7	0.598	4.8 ± 3.3	4.8 ± 2.4	0.987
Mood	7.6 ± 2.7	8.4 ± 2.3	0.241	2.7 ± 2.9	3.0 ± 3.0	0.812	4.9 ± 3.5	5.3 ± 3.0	0.680
Walking	2.9 ± 3.9	2.5 ± 3.8	0.913	0.6 ± 1.9	0.8 ± 2.2	0.795	2.4 ± 3.8	1.7 ± 3.3	0.987
Work	5.6 ± 3.1	6.2 ± 3.3	0.492	2.3 ± 2.7	2.3 ± 2.7	0.988	3.3 ± 2.8	3.9 ± 3.1	0.552
Relationship	6.6 ± 2.6	7.0 ± 2.8	0.475	2.1 ± 2.6	3.1 ± 2.6	0.193	4.5 ± 3.2	3.9 ± 2.8	0.509
Sleep	4.8 ± 3.3	5.5 ± 3.9	0.400	2.1 ± 2.8	1.5 ± 2.4	0.581	2.7 ± 2.8	4.0 ± 3.4	0.243
Enjoyment of life	7.4 ± 2.3	8.2 ± 1.4	0.318	2.9 ± 3.1	3.3 ± 2.3	0.391	4.6 ± 3.1	5.0 ± 2.1	0.631
Eat	6.0 ± 3.0	6.9 ± 3.7	0.176	2.4 ± 3.0	3.04 ± 3.0	0.505	3.6 ± 3.1	3.9 ± 3.2	0.825
Touch your face	5.9 ± 3.4	7.6 ± 3.1	0.034	3.7 ± 3.1	3.1 ± 3.2	0.585	1.9 ± 4.6	3.4 ± 4.5	0.315
Brush your teeth	5.6 ± 3.5	6.5 ± 4.0	0.281	3.1 ± 3.1	3.5 ± 3.2	0.779	2.6 ± 2.6	3.1 ± 3.2	0.626
Smile or laugh	5.2 ± 3.6	5.1 ± 4.0	0.945	2.7 ± 2.9	2.6 ± 3.1	0.830	2.5 ± 3.1	2.5 ± 3.0	0.944
Talk	5.6 ± 3.8	6.1 ± 3.8	0.572	1.8 ± 2.3	3.5 ± 2.9	0.058	3.7 ± 3.7	2.6 ± 3.0	0.294
Open your mouth wide	5.7 ± 3.5	5.3 ± 4.1	0.858	2.6 ± 2.4	2.6 ± 3.2	0.712	3.2 ± 3.0	2.7 ± 3.0	0.538
Eat hard food	7.5 ± 2.6	8.4 ± 2.4	0.142	4.2 ± 3.8	5.0 ± 3.1	0.573	3.3 ± 3.4	3.4 ± 2.3	0.601
Worst NRS	9.3 ± 1.7	9.6 ± 1.0	0.985	4.7 ± 3.4	5.2 ± 2.7	0.650	4.7 ± 3.8	4.4 ± 2.8	0.956
Least NRS	5.3 ± 2.2	6.0 ± 2.1	0.297	2.1 ± 1.9	2.3 ± 2.1	0.835	3.2 ± 2.8	3.7 ± 2.8	0.271
Mean NRS	7.4 ± 1.5	7.9 ± 1.4	0.293	3.1 ± 2.3	3.5 ± 2.3	0.600	4.3 ± 2.7	4.3 ± 2.4	0.985
Pain now	4.4 ± 3.6	5.5 ± 2.7	0.358	1.0 ± 2.2	1.7 ± 2.5	0.185	3.4 ± 3.5	3.8 ± 2.4	0.450

**Table 5:** The distribution of the PGIC scores in each group.

	PGIC 1/7	PGIC 2/7	PGIC 3/7	PGIC 4/7	PGIC 5/7	PGIC 6/7	PGIC 7/7
Type 1 TN	0	0	2	0	1	11	4
Type 2 TN	0	0	2	1	2	15	2

## Data Availability

Data will be available on reasonable request.
